# Posterolateral plating increases peroneal tendon thickness without affecting clinical outcomes: a ultrasonographic comparative study of 76 SER ankle fractures

**DOI:** 10.1007/s00590-026-04736-8

**Published:** 2026-04-24

**Authors:** Pedro José Labronici, Jonas Pessoa de Campos, João Pedro de Araújo Almeida, William Dias Belangero, Gustavo Waldolato, Robinson Esteves Pires, Antônio Tufi Neder Filho, Túlio Vinícius de Oliveira Campos, David Rojas, Anderson Freitas, Vincenzo Giordano, Alexandre Leme Godoy-Santos

**Affiliations:** 1 Fluminense Federal University, Niterói, Brazil; 2 Medicine School of Petrópolis, Petrópolis, Brazil; 3 Santa Teresa Hospital of Petrópolis, Petrópolis, Brazil; 4 State University of Campinas, Campinas, Brazil; 5 Department of Orthopedics and Traumatology, Faculty of Medical Sciences of Minas Gerais, Belo Horizonte, Brazil; 6 Department of the Locomotor System, Federal University of Minas Gerais, Belo Horizonte, Brazil; 7 Hospital for Special Surgery, New York, USA; 8 HOME Hospital, Brasília, Brazil; 9 Professor Nova Monteiro Service, Miguel Couto Hospital, Rio de Janeiro, Brazil; 10 Institute of Orthopedics and Traumatology, University of São Paulo, São Paulo, Brazil

**Keywords:** Ankle fracture, Distal fibula fracture, Lateral plate, Posterolateral plate

## Abstract

**Objectives:**

To evaluate, using ultrasonography and the AOFAS (The American Orthopaedic Foot and Ankle Society) score, changes in the peroneal tendons of patients with ankle fractures resulting from a supination-external rotation (SER) mechanism treated with either lateral or posterolateral plate fixation.

**Methods:**

This retrospective observational study was conducted at a tertiary level III trauma center. Adult patients (> 18 years) with unilateral SER ankle fractures, a minimum follow-up of 1 year, no persistent symptoms, and fixation using a 1/3 tubular 3.5-mm locking plate (lateral or posterolateral) were included. Exclusion criteria comprised bilateral injuries, Weber A/C fractures, pathological fractures, previous ankle disorders, infection, postoperative pain, or functional limitations. The primary outcomes were peroneal tendon thickness on ultrasonography and functional performance assessed using the AOFAS Ankle-Hindfoot Scale. Outcomes were compared between fixation techniques.

**Results:**

Seventy-six patients were included (65.8% aged 35–64 years, 60.5% women, 53.9% left-sided injuries). In most cases (71.1%), tendon thickness was greater on the operated side. Posterolateral plating demonstrated a higher frequency of positive tendon-thickness differences. Most differences measured 0–2 mm (56.6%), with a statistically significant difference between lateral and posterolateral fixation groups (*p* < 0.001). AOFAS scores were high in both groups, indicating satisfactory postoperative functional outcomes.

**Conclusions:**

A difference in peroneal tendon thickness between the operated and contralateral sides was observed in 89.5% of patients, and these differences were significantly greater after posterolateral plating. Although plate position directly influenced postoperative tendon thickness, no clinically relevant consequences were identified.

***Level of evidence*:**

Level III.

## Introduction

Ankle fractures are the third most common fracture in older adults, after hip and distal radius fractures [1–4]. Danis-Weber type B lateral malleolar fractures are the most common fractures due to the supination–external rotation (SER) mechanism, an injury characterized by an obliquely shaped fracture line which propagates in the anteroinferior to posterosuperior direction [5]. In these displaced fractures, restoration of the anatomic length and rotation of the distal fibula is necessary, usually requiring open reduction internal fixation [6–9].

The standard method used to treat these fractures is lag-screw fixation with a lateral neutralization plate. The antiglide plate technique was advocated by Brunner and Weber [10] to overcome some of the disadvantages of lateral plating, such as surgical wound complications or pain caused by the subcutaneous position of the plate [11], sural nerve injury [12], and risk of screw penetration into the joint space. This construct was found to be biomechanically stronger than standard lateral plating and has been advocated for use in osteoporotic bones [13]. Although the posterolateral technique offers improved biomechanical stability, Weber and Kraus [14] reported high complication rates, with 30% of patients experiencing peroneal tendon injuries and 43% requiring removal of the implant.

We hypothesized that the posterolateral plate could change the morphology of the peroneal tendons, therefore compromising clinical outcomes. The aim of the present study was to evaluate, using ultrasonography and the AOFAS score/Ankle-Hindfoot Scale, changes in the peroneal tendon in patients with ankle fractures due to SER mechanisms treated with two plate fixation techniques.

## Methods

This was a single-center, retrospective, observational study conducted in a tertiary-referral level III trauma center including 76 patients who sustained unilateral fractures resulting from supination and external rotation of the ankle. Patients treated from 2000 to 2023 were included in the study sample. These patients were divided into two groups: Group 1 with lateral malleolar plate, and Group 2 with a posterolateral plate. The same access route was used in both techniques, with maximum preservation of the peroneal tendons, especially during the placement of the plates in a posterolateral position, avoiding mobilization, retraction, and dissection of the peroneal tendons.

**Fig. 1:**
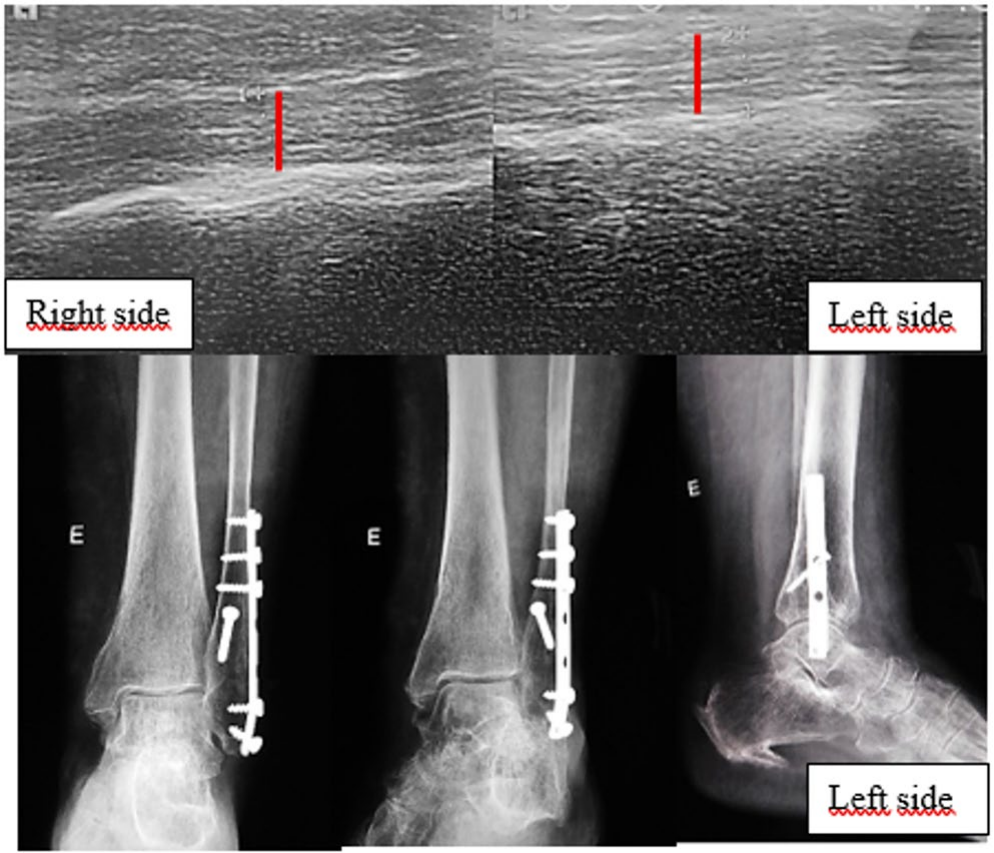
Ultrasound and plain radiographs of a left ankle fracture comparing the injured and contralateral tendons

**Fig. 2:**
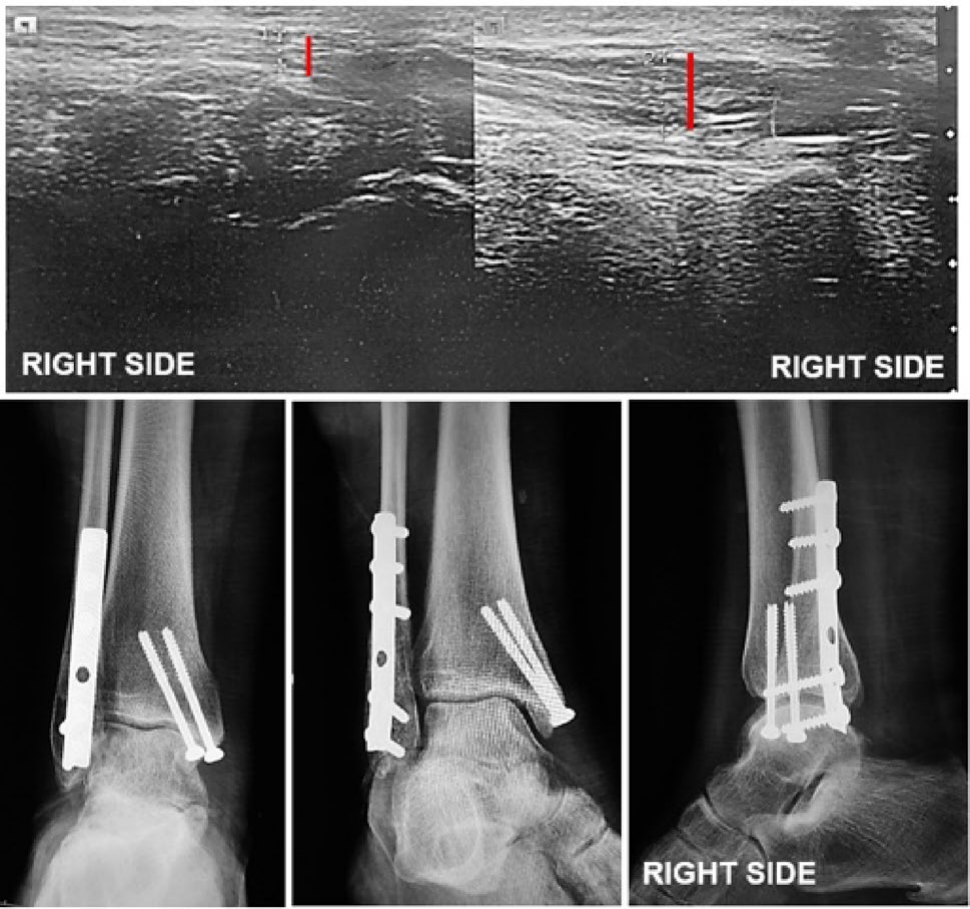
Ultrasound and plain radiographs of a right ankle fracture comparing the injured and contralateral tendons

Examination of the peroneal tendons with ultrasonographic evaluations were performed at a minimum 1-year postoperative follow-up and was performed using a Siemens Healthineers Acuson NX3 ultrasonography system (2018) with a VF12-4 linear transducer (frequency range 6.7–9.4 MHz), placed longitudinally and perpendicular to the lateral malleolus, using the standard preset for musculoskeletal and soft tissues, with the ankle at 90° to the plane of the examination table and with the foot in physiological neutral alignment, with the patient relaxed. To confirm the transducer was perpendicular to the examination table, it was supported on a 35 × 20 × 5 cm expanded polystyrene block with all of its surfaces flat and squared. Three images were obtained, 30 s apart, after making sure that the patient’s ankle and foot were completely relaxed. All patients had both ankles scanned for comparative analysis (Figs. 1 and 2).

Tendon thickness was measured at the lower portion of the plate attached to the fibula. Clinical assessment was based on the AOFAS Ankle-Hindfoot Scale, as translated and validated for use in Brazilian Portuguese [15], and absence of any complaints 1 year after surgery.

The inclusion criteria were age > 18 years, fractures resulting from SER, a duration of follow-up > 1-year, unilateral fracture, absence of any complaints, and osteosynthesis performed with a one-third (1/3) tubular 3.5 mm locking plate placed in the posterolateral or lateral region of the fibula.

Exclusion criteria comprised bilateral injuries, Weber A/C fractures, tri-malleolar fractures, pathological fractures, previous ankle disorders, infection, postoperative pain, or functional limitations. The difference between tendon thickness on the injured (operated) and contralateral sides after surgery was analyzed and categorized qualitatively as positive, absent, or negative (i.e., tendon thicker on the operated side, no difference in thickness, or tendon thicker on the contralateral side), as well as divided into quantitative classes: Class I, < 1 cm; Class II, 1–1.9 cm; Class III, 2–2.9 cm; and Class IV, > 3 cm.

Descriptive analysis was based on frequency distributions and calculation of descriptive statistics (proportions of interest, minimum, maximum, mean, median, standard deviation, and coefficient of variation [*CV*]). As noted above, the difference in thickness between the operated and contralateral tendons was classified as follows: Class I, < 1 cm; Class II, 1–1.9 cm; Class III, 2–2.9 cm; and Class IV, > 3 cm.

For inferential analysis of qualitative variables, the frequency distributions of two independent groups were compared using the chi-square test. For inferential analysis of quantitative variables, the distributions of a quantitative or ordinal variable of two independent groups were compared by Student’s *t*-test if normally distributed or by the Mann–Whitney *U* test otherwise.

Correlation analysis between the difference in tendon thickness and other quantitative variables was performed through visual inspection of a scatter plot of the two variables, obtaining the line of best fit and calculating the coefficient of determination $\:{R}^{2}$, given by the square of Pearson’s linear correlation coefficient $\:\left(r\right).$ Significance was accepted at the 5% level (*P* ≤ 0.05).

## Results

This study analyzed 76 surgical cases, including 49 treated with lateral plates and 27 with posterolateral plates, with a statistically significant difference in group distribution (*P* = 0.016).

The most prevalent characteristics were female predominance (60.5%), left-sided injury (53.9%), age between 35 and 64 years (65.8%), and surgery performed less than 6 years before the time of assessment (89.5%).

Table 1 summarizes age and postoperative time for the total sample and by fixation type. Age showed moderate variability and normal distribution (*P*  > 0.05), allowing comparison by Student’s t-test, while time since surgery was highly variable and non-normal (*P* < 0.05), requiring the Mann–Whitney U test. Mean age was 50.4 years, and mean time since surgery was 2.7 years, with no significant differences between fixation groups.

**Table 1:** Distribution according to age and surgical time of patients, in the total sample and in subgroups defined according to the positioning of the fibula plate. *Source*: Authors, 2026

Variable	Sample	Minimum	Maximum	Median	Mean	SD	CV	*p*-value *
Age (years)	Total	20.0	87.0	52.5	50.4	15.5	0.31	0.576
	L plate	20.0	81.0	50.0	48.4	16.1	0.33	0.654
	PL plate	26.0	87.0	56.0	52.6	14.7	0.28	0.506
Time after surgery (years)	Total	1.0	15.0	1.5	2.7	3.3	1.22	< 0.001
	L plate	1.0	15.0	1.6	2.6	3.2	1.24	< 0.001
	PL plate	1.0	13.0	1.5	2.8	3.4	1.22	< 0.001

L, lateral; PL, posterolateral; SD, standard deviation; CV, coefficient of variation

*Shapiro-Wilk normality test

Table 2 shows that, in most cases (71.1%), the difference between the tendons was positive, indicating greater tendon thickness on the operated side. This trend was observed in both groups, but the posterolateral plating group had significantly more cases of positive difference between the tendons and fewer cases of null or negative difference. The difference in tendon difference classifications (negative, null, positive) between plate types was statistically significant (*P* = 0.006).

**Table 2:** Distribution of the difference between tendons observed after surgery, in the overall sample and in subgroups determined according to plate location. *Source*: Authors, 2026

Difference between tendons	Total Sample (*n* = 76)		L plate (*n* = 49)		PL plate (*n* = 27)		*p*-value
	F	%	F	%	F	%	
Negative	14	18.4	12	24.5	2	7.4	0.006 ^(a)^
Null	8	10.5	8	16.3	0	0	
Positive	54	71.1	29	59.2	25	92.6	
Class IV Negative (3.0–3.9 cm)	23	30.3	17	34.7	1	3.7	**<** 0.001^(b)^
Class III Negative (2.0–2.9 cm)	20	26.3	9	18.4	0	0	
Class II Negative (1.0–1.9 cm)	8	10.5	2	4.1	0	0	
Class I Negative (< 1.0 cm)	3	3.9	1	2.0	1	3.7	
Class 0 (Null difference)	8	10.5	8	16.3	0	0	
Class I Positive (< 1.0 cm)	1	1.3	0	0	6	22.2	**< **0.001^(b)^
Class II Positive (1.0–1.9 cm)	0	0	0	0	1	40.7	
Class III Positive (2.0–2.9 cm)	0	0	0	0	6	22.2	
Class IV Positive (3.0–3.9 cm)	13	17.1	12	24.5	2	7.4	
Class 0 (Null difference)	8	10.5	8	16.3	0	0	
Negative difference between tendons							n.a.
Class I Negative (< 1.0 cm)	13	92.9	12	100.0	1	50.0	
Class IV Negative (3.0–3.9 cm)	1	7.1	0	0	1	50.0	
Positive difference between tendons							
Class I Positive (< 1.0 cm)	23	42.6	17	58.6	6	24.0	0.007^(b)^
Class II Positive (1.0–1.9 cm)	20	37.0	9	31.0	1	44.0	
Class III Positive (2.0–2.9 cm)	8	14.8	2	6.9	6	24.0	
Class IV Positive (3.0–3.9cm)	3	5.6	1	3.4	2	8.0	

L, lateral; PL, posterolateral; F, frequency; %, percentage; n.a., not applicable

^(a)^ Chi-square test

^(b)^ Mann-Whitney test

Most differences between tendons were in the 0–2 cm range (56.6% of cases in the overall sample), with a significant difference between plate groups (*P* < 0.001). When considering the absolute difference between the tendons, i.e., only the magnitude of the difference between the sides, the 0–2 cm range was even more frequent (73.7%), again with a significant difference between plate fixation groups (*P* < 0.001).

Of the 14 cases of negative difference between tendons, the majority (92.9% of class I) exhibited < 1 cm difference, and only one case (7.1% of class IV) had a difference > 3 cm. As the posterolateral plate group had only 2 cases with a negative difference, no between-group comparison was done. Among the 54 cases of positive difference between tendons, the majority (79.6% of the overall sample) exhibited a 0–2 cm difference, 14.8% between 2 and 3 cm (class III), and 5.6% > 3 cm (class IV). There was a significant difference in the distributions of positive tendon differences between the two technique groups (*P* < 0.007). The occurrence of negative differences between tendons was not compared, because there were only two such cases in the posterolateral plating group. Overall and in the two technique groups, the coefficients of variation showed high variability.

Table 3 presents key AOFAS score statistics for the sample overall and for the two groups defined by plate location. In all groups, high mean and median scores were observed, indicating good postoperative quality of life. The coefficients of variation were consistent with low variability between patients. Between-group comparison performed using the Mann–Whitney *U* test did not identify a statistically significant difference in terms of AOFAS scores (*P*  = 0.492).

**Table 3:** AOFAS score on the total sample and on subgroups defined according to the position of the plate. *Source*: Authors, 2026

Variable	Minimum	Maximum	Median	Mean	SD	CV	*p*-value *
Total	65	100	88.5	89.4	9.3	0.10	
L plate	75	100	90.00	90.2	8.4	0.09	0.492
PL plate	65	100	88.0	88.0	10.6	0.12	

L, lateral; PL, posterolateral; SD, standard deviation; CV, coefficient of variation

*Mann-Whitney test

## Discussion

The present study demonstrated a statistically significant difference in peroneal tendon thickness, as assessed by ultrasonography, between patients who had undergone osteosynthesis with a lateral plate and those who had received a posterolateral plate. Although tendon thickness was greater in the posterolateral group, this did not translate into clinically relevant repercussions, as AOFAS scores remained comparable and showed no indication of symptomatic peroneal tendonitis.

For the surgical treatment of fractures of the distal fibula, different locations are available for plate placement, such as on the lateral versus the posterolateral side of the lateral malleolus. Although the lateral location is most common in practice, studies have shown that a posterolateral location offers greater mechanical properties. Nevertheless, posterolateral plating still raises concerns due to the risk of injury to the peroneal tendons [16]. However, several pathological conditions can mimic the signs and symptoms of peroneal tendinopathy, representing an important differential diagnosis. These include sprains of the lateral ankle ligaments, fractures of the lateral process of the talus, ankle impingement syndrome, injuries of the distal tibiofibular syndesmotic ligaments, osteochondral lesions of the talar dome, subtalar arthropathy, os trigonum syndrome, sural nerve neuropathy, and anterior calcaneal process fractures [17]. Posterior and posterolateral plating of lateral malleolus fractures has been associated with varying complication rates, especially regarding peroneal tendinopathy. Ostrum [18], reported 4 cases (12.5%) of transient peroneal tendinopathy. Likewise, Treadwell and Fallat [19] reported a 2.81% incidence (2/71 cases) of peroneal tendinopathy after posterolateral plate fixation.

Comparative studies, such as those conducted by Lamontagne et al. [20] and Wissing et al. [21], did not identify statistically significant differences. However, the findings of Weber and Krause [14] contradict these results: the authors found evidence of peroneal tendonitis in 30 of 70 patients (43%) who underwent posterolateral plating, all of whom developed persistent discomfort and required hardware removal. In a review of 93 patients treated with posterolateral plate fixation, Winkler and Weber [22] reported 66.7% excellent, 27.9% good, and only 5.4% unsatisfactory results. Poor outcomes were all observed in patients over 55 years of age and were mostly associated with pre-existing osteoarthritis. Elwahab et al. [23], showed that both groups achieved good clinical, radiographic, and functional outcomes, with no statistically significant difference (*P* > 0.05). However, a trend towards better outcomes was observed in the posterolateral plating group (95% good to excellent vs. 80%). Ahn et al. [24], in an observational study of 70 patients treated for lateral malleolar fractures resulting from SER mechanisms, demonstrated a low rate of clinical symptoms consistent with tendinopathy (4.3%) and they did not identify any gross involvement of the tendons or tendon sheath during intraoperative inspection. Furthermore, the authors relied primarily on clinical findings suggestive of peroneal tendinopathy. These findings corroborate those of Treadwell and Fallat [19], and Kilian et al. [25], in a prospective study, reiterated the effectiveness posterolateral plating. As demonstrated in previous studies, functional outcomes were equivalent between the lateral and posterolateral fixation groups, with no statistically significant difference in AOFAS scores after 1 year of follow-up (*P* = 0.37), which is consistent with our results. The posterolateral plating group also showed a trend towards a lower complication rate (15% vs. 29.3%), although this difference failed to reach statistical significance (*P* = 0.31).

When properly positioned, the posterolateral plate offers satisfactory biomechanical stability with an acceptable complication profile, providing a safe alternative to conventional lateral fixation, especially in unstable ankle fractures. Our study evaluated the difference in peroneal tendon thickness between the operated side and the contralateral side in patients undergoing fixation with lateral or posterolateral plating. In both groups, most cases had greater tendon thickness on the operated side, i.e., a positive difference (71.1%). A statistically significant difference (*P* = 0.006) between the lateral and posterolateral groups was observed regarding type of difference in tendon thickness (negative, null, or positive), suggesting an association between plate positioning and subsequent change in tendon thickness, which also contradicts the findings of Weber and Krause [14]. 

Analysis of distributions showed that the group treated with posterolateral plating had a significantly higher proportion of increased tendon thickness in the operated side (positive difference), a lower number of cases with no appreciable difference, and an even lower proportion of cases with reduced thickness in the operated side (negative difference) when compared to the lateral plating group.

In both groups the majority of cases had < 2 cm difference between the operated and contralateral sides (56.6%), although the difference between the groups was significant (*P*  < 0.001). Considering the absolute measurement of this difference (i.e., only the magnitude of the asymmetry between tendons, regardless of which side was thicker), the majority of cases were also in the 0–2 cm range (73.7%), again with a statistically significant difference between the two groups (*P* < 0.001).

Of the 14 cases with a negative difference, 92.9% showed a thickness reduction of < 1 cm in the operated side relative to the contralateral tendon (Class I), while only one case (7.1%) showed a difference > 3 cm (Class IV). As only two of these cases occurred in the posterolateral plating group, an adequate comparison of this subcategory between groups could not be conducted.

Among the 54 cases with a positive difference, 79.6% showed an increase in thickness of < 2 cm (Class I and II); 14.8% showed an increase of 2–3 cm (Class III); and in only 5.6% was the increase in thickness > 3 cm in relation to the contralateral side (Class IV). There was a significant difference in the distributions of these positive differences between the two technique groups (*P* < 0.007).

**Fig. 3:**
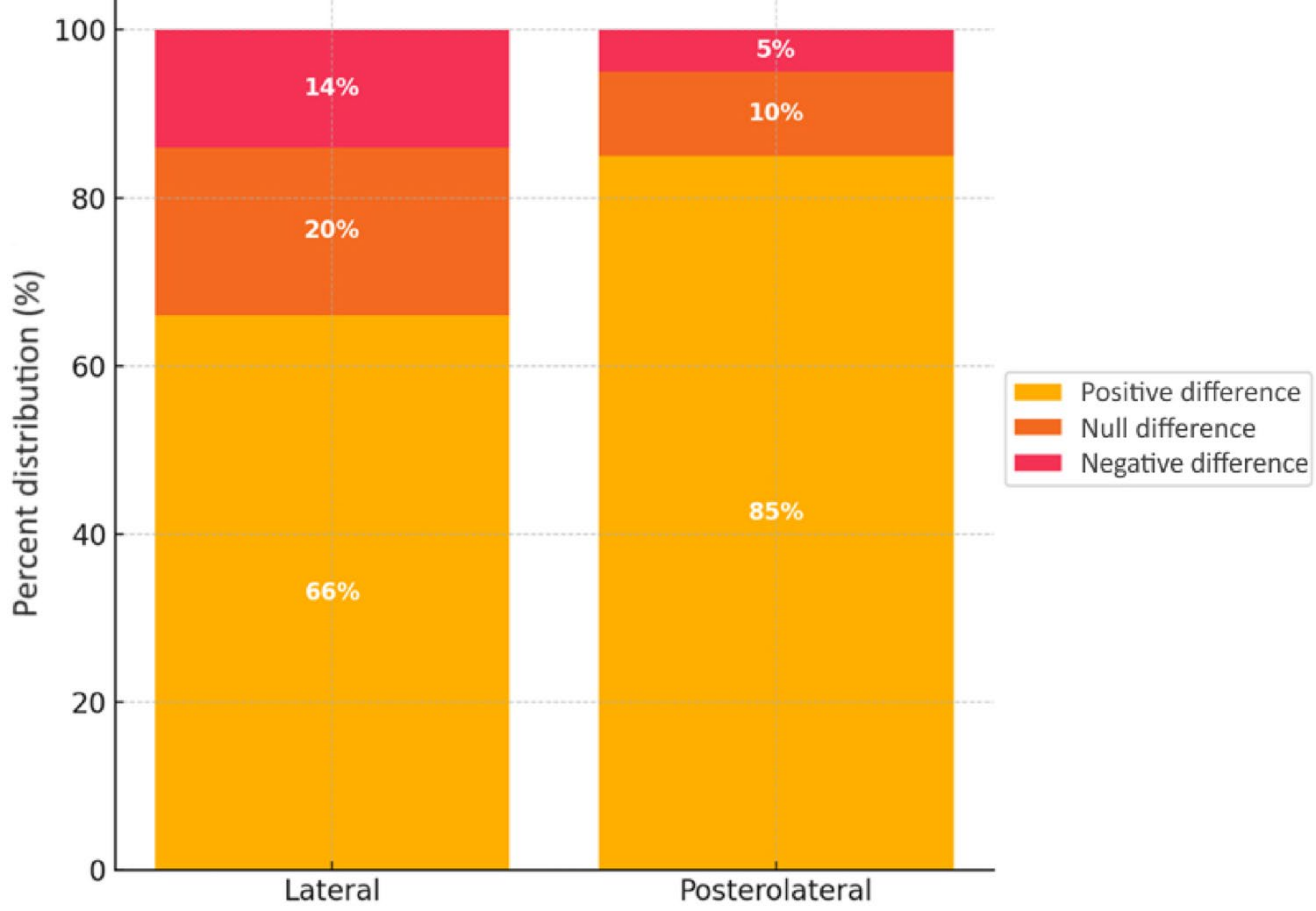
Classification of the difference in thickness of the peroneal tendon by plate location

In summary, the difference in peroneal tendon thickness between the repaired and contralateral sides typically remains < 2 cm. However, all comparisons performed indicated a statistically significant association between plate location (lateral or posterolateral) and change in tendon thickness, whether by classification (*P* = 0.006), direct measurement (*P*  < 0.001), absolute measurement (*P*  < 0.001), or when analyzing only positive differences (*P*  = 0.007). These findings suggest that plate positioning directly influences morphological changes in the peroneal tendon (Fig. 3).

This study has some limitations. The retrospective observational design is subject to selection and information biases. Nevertheless, all ultrasound scans were performed by a single examiner, which provides greater consistency and reproducibility in measurements. Although relatively small, the sample was homogeneous in terms of clinical profile and surgical methods used. The absence of preoperative ultrasonographic assessment represents a limitation of this study; however, obtaining a reliable and standardized ultrasonographic examination in the setting of an acute ankle fracture is often challenging due to pain, swelling, and limited patient cooperation.

Despite these limitations, the results reported herein provide further evidence of the safety of posterolateral plating, which was associated with a slight increase in thickness of the peroneal tendon that had no significant clinical repercussions.

## Conclusions

In this cohort of patients with SER-type ankle fractures, posterolateral plating was consistently associated with greater postoperative peroneal tendon thickness when compared with lateral plating. Although this morphological alteration was statistically significant across all analytical approaches, it did not translate into functional impairment, as reflected by uniformly high AOFAS scores. These findings suggest that, while plate position influences tendon morphology, the observed thickening likely represents an adaptive or benign postoperative response rather than a clinically meaningful complication. Posterolateral plating therefore remains a biomechanically reliable and clinically safe option for the fixation of unstable distal fibula fractures, provided that meticulous surgical technique and awareness of regional tendon anatomy are maintained. Future prospective studies with long-term functional and imaging follow-up are warranted to clarify whether these morphological changes carry any implications beyond the medium-term outcomes observed in this study.

## Data Availability

The data that support the findings of this study are available from the corresponding author, upon reasonable request.
